# THE LEAD GUIDE: LIFE PLANNING IN EARLY ALZHEIMER’S AND DEMENTIA

**DOI:** 10.1093/geroni/igz038.004

**Published:** 2019-11-08

**Authors:** Kara B Dassel, Katherine Supiano, Rebecca Utz, Sara Bybee, Eli Iacob

**Affiliations:** University of Utah, Salt Lake City, Utah, United States

## Abstract

To address the characteristics of Alzheimer’s disease and related dementias (ADRD) that complicate end-of-life (EOL), we created and validated a dementia-specific EOL planning instrument. This instrument can be used to facilitate discussions and provide documentation of EOL values and care preferences prior to loss of decisional capacity. Instrument development used a mixed-method design that included: a) conducting a series of focus groups (healthy adults, persons with early-stage ADRD, and dementia caregivers) to develop and confirm content, comprehensiveness, and usability, b) evaluation by content experts to verify instrument utility in clinical practice; and c) conducting a national survey of healthy older adults and adults with early-stage ADRD to evaluate instrument psychometric properties. We describe the expansion of the instrument from survey tool into a user-friendly guide: “The LEAD Guide” or Life-Planning in Early Alzheimer’s and Dementias Guide. We describe the utility of the LEAD Guide for persons planning ahead for the possibility of ADRD, those with MCI and their families, for providers to use with patients and families, and for researchers studying the EOL preferences and values of persons in these populations. Instructions specific to each user group are health literacy appropriate, and include a glossary of terms. Age-friendly graphic design and availability in both print and e-versions enhance utility. The LEAD Guide can be utilized to help inform EOL care decisions and ensure that they align with the patient’s values and may serve as the basis of an intervention to support choice in persons with dementia.

